# A nomogram for predicting the readmission within 6 months after treatment in patients with acute coronary syndrome

**DOI:** 10.1186/s12872-022-02873-6

**Published:** 2022-10-26

**Authors:** Dongyun Li, Ying Lin, Wenjing Dong, Yalei Hu, Ke Li

**Affiliations:** 1 Department of the First Health Care, the Second Medical Center of People’s Liberation Army General Hospital, 100853 Beijing, P. R. China; 2 Department of Cardiology, Hainan Hospital of Chinese People’s Liberation Army General Hospital, 572013 Sanya, Hainan Province P. R. China; 3 Department of Geriatric Medicine, Hainan Hospital of Chinese People’s Liberation Army General Hospital, 572013 Sanya, Hainan Province P. R. China; 4 Department of Hematology, Hainan Hospital of Chinese People’s Liberation Army General Hospital, 572013 Sanya, Hainan Province P. R. China; 5 Jianglin Road, Haitang District, 572013 Sanya City, Hainan Province P. R. China

**Keywords:** Acute coronary syndrome, Readmission, Prediction model

## Abstract

**Purpose:**

To explore predictors for readmission within 6 months of ACS patients, and to build a prediction model, and generate a nomogram.

**Methods:**

The retrospective cohort study included 498 patients with ACS in the Second Medical Center of the Chinese People’s Liberation Army General Hospital between January 2016 and March 2019. Univariate and multivariate logistic regression with odds ratios (OR) and two-sided 95% confidence interval (CI) analysis were used to investigate predictors for readmission within 6 months. The cohort was randomly divided into training cohort to develop a prediction model, and the validation cohort to validate the model. The receiver operating characteristic curve (ROC) and the calibration curve was used to assess discriminative power and calibration.

**Results:**

Eighty-three ACS patients were readmitted within six months, with a readmission rate of 16.67%. Predictors included ACS type, treatment, hypertension, SUA, length of stay, statins, and adverse events occurred during hospitalization were used to form a six-month readmission prediction model for readmission within 6 months in ACS patients. The area under the curve (AUC) of the model was 0.788 (95%CI: 0.735–0.878) and 0.775 (95%CI: 0.686–0.865) in the training cohort and the validation cohort, respectively. Calibration curves showed the good calibration of the prediction model. Decision-curve analyses and clinical impact curve also demonstrated that it was clinically valuable.

**Conclusion:**

We used seven readily available predictors to develop a prediction model for readmission within six months after treatment in ACS patients, which could be used to identify high-risk patients for ACS readmission.

**Supplementary Information:**

The online version contains supplementary material available at 10.1186/s12872-022-02873-6.

## Introduction

Acute coronary syndrome (ACS) is a group of clinical syndromes based on the rupture or invasion of coronary atherosclerotic plaque followed by complete or incomplete occlusive thrombosis [1]. According to the World Health Organization, ACS causes significant morbidity and mortality, which is associated with 126 deaths per 100,000 people globally [2]. Early readmission after ACS is associated with poor patient outcomes,which would increase the mortality [3]. Besides, Readmission increases the time, energy, and economic burden of patients and their families and leads to adverse consequences such as shortage of department beds, increased medical service costs, and decreased efficiency of medical resource utilization [4]. Therefore, identifying modifiable factors associated with ACS readmission could help provide preventive interventions that improve outcomes and save healthcare costs.

Although previous studies have found comorbidities and smoking are risk factors for readmission [5], predictor models for the readmission of ACS were scarce. Besides, current research mainly focused on the prognostic factors of a specific type of ACS [6], or a single predictor of short-term or long-term prognosis of ACS, such as decreased glomerular filtration rate (GFR) and increased urinary micro-protein/creatinine ratio [7], the level of mean platelet volume [8], etc. Albuquerque RN et al. [9] proposed a prediction model for ACS hospital readmission, but the model could not be used for predicting the probability of readmission for an individual patient [10]. What is more, current researches mainly focused on the risk factors of 30-day or one-year readmission and the model to predict readmission within 6 months is rare, which is also account for a large proportion of early readmissions according to previous studies and our observation [11–14]. Hence, a prediction model, especially a nomogram, is a useful tool for predicting readmission risk within 6 months in ACS patients to improve personalized decision-making needs to be developed.

This study of readmissions in patients with ACS treatment within 6 months included an analysis of predictors derived from data on clinical characteristics, laboratory tests, comorbidities, and medication. The clinical results were used to establish a predictive model for screening high-risk groups, aiming to provide a theoretical basis for early prevention and improved prognosis of ACS patients treated for readmission.

## Methods

### Study population

**Fig. 1:**
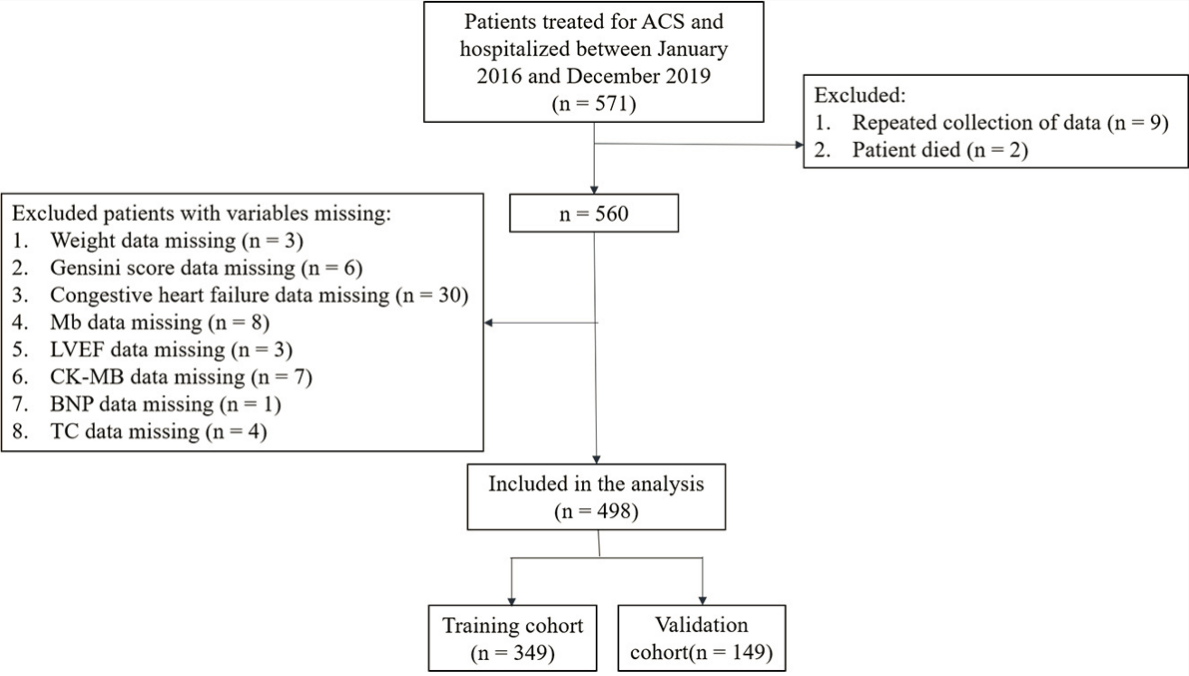
Flowchart of the study population selection. ACS: acute coronary syndrome; Mb: myoglobin; LVEF: left ventricular ejection fraction; CK-MB: creatine kinase-MB; BNP: brain natriuretic peptide; TC: total cholesterol

This retrospective cohort study was based on the patients hospitalized in the Second Medical Center of the Chinese People’s Liberation Army General Hospital. Patients treated for ACS and hospitalized between January 2016 and March 2019 were included in the study. This study was approved by the Ethics Committee of the Second Medical Center of the Chinese People’s Liberation Army General Hospital and written informed consent was obtained from each patient in accordance with institutional guidelines. All methods were carried out in accordance with relevant guidelines and regulations. Inclusion criteria were as follows: (1) Patients aged ≥ 18 years; (2) patient was diagnosed with ACS for the first time and was discharged after treatment; (3) the baseline data, laboratory examination data and imaging examination data of the patients were complete. Exclusion criteria included any one of the following: (1) patients had organic mitral valve disease (rheumatic heart disease, hypertensive heart disease, mitral valve calcification, etc.); (2) patients who died during initial hospitalization or follow-up. Based on the inclusion and exclusion criteria, there were 551 ACS patients in our study. Figure 1 shows the flow chart of our study.

### Outcome variable

The outcome was readmission events within 6 months after treatment in patients with ACS. The follow-up duration was 6 months after treatment by telephone and re-examination. The end point of follow-up was the occurrence of readmission events or the follow-up period ended on September 12, 2019.

### Data collection

The information about demographic characteristics, clinical characteristics, complications, laboratory indexes, and medical treatment were collected.

Demographic characteristics include age, gender, smoking history, alcohol drinking history. Height (m), weight (kg), body mass index (BMI, kg/m^2^), Gensini score, ACS type [non-ST-segment elevation myocardial infarction (NSTEMI), ST-segment elevation myocardial infarction (STEMI) and unstable angina (UA)], number of diseased vessels, and treatment [percutaneous coronary intervention (PCI) and others (cardiovascular primary prevention, cardiovascular secondary prevention, thrombolysis, drugs)] were collected clinical characteristics. Histories of hypertension, diabetes mellitus, and other diseases (gout, congestive heart failure, end-stage renal disease, chronic obstructive pulmonary disease, peripheral vascular disease, hypothyroidism, depression, etc.) were the studied complications for all the patients.

Serum biomarkers included hemoglobin (HGB, g/L), counts of white blood cells (WBC, 10^9^/L), blood platelet (PLT, 10^9^/L), neutrophils (NEUT, 10^9^/L), creatinine (Cr, µmol/L), serum uric acid (SUA, µmol/L), glomerular filtration rate (eGFR, ml/min), creatine kinase-MB (CK-MB, µg/L), and brain natriuretic peptide (BNP, pg/mL) at first admission. Blood lipid was monitored, including baseline levels of total cholesterol (TC, mmol/L), triglyceride (TG, mmol/L), high-density lipoprotein cholesterol (HDL, mmol/L), and low-density lipoprotein cholesterol (LDL, mmol/L) at first admission.

Medications including clopidogrel, calcium channel blockers, angiotensin-converting enzyme inhibitors/angiotensin receptor blockers (ACEI/ARB), statins, and diuretics were collected during the first hospitalization and within 6 months after discharge.

The length of stay and whether adverse events occurred during hospitalization were collected at discharge. Adverse events included cardiac arrest, coronary dissection, coronary perforation, acute kidney injury, major bleeding, cardiogenic shock, acute respiratory failure, etc.

### Establishment and validation of the predictive model

Using univariate analysis, the differences between the readmission group and the non-readmission group were compared and the significance of each variable was assessed. Variables with *P*<0.05 in univariate analysis were considered potential candidates and included in multivariate logistic regression analysis to investigate predictors for readmission within 6 months after treatment in ACS patients. Two multivariate logistic regression models have developed in the multivariate logistic regression analysis with odds ratios (OR) and two-sided 95% confidence interval (CI), Model 1 was the crude model, and Model 2 adjusted the age, BMI, gender, smoking history, drinking history and Gensini score. The eligible patients were randomly divided into the training cohort (70%, n = 349) for prediction model development, and the validation cohort (30%, n = 149) to validate the model’s predictive performance and discriminative power. The logistic regression prediction model was developed using predictors explored in multivariate logistic regression analysis, and a nomogram was drawn. The receiver operating characteristic curve (ROC) and calibration curve, the value of area under the curve (AUC), accuracy, specificity, and sensitivity were used to assess discriminative power and calibration. Finally, the model was internally validated via bootstrapping resampling of the construction data set (with 1000 bootstrap samples per model) to obtain optimism corrected discrimination via the C-index for rebleeding. Besides, decision-curve analysis (DCA) and clinical impact curve were also used to determine the clinical net benefit associated with the use of the model.

### Statistical analysis

We conducted descriptive analysis for the studied variables. The normally distributed data were described as mean ± standard deviation (Mean ± SD), and the independent samples t-test was used for comparison between groups; non-normal data were described as the median and interquartile range [M (Q1, Q3)], the Mann-Whitney U test was used for comparison between groups. Categorical variables were described by the number of cases and percentages [n (%)], and the comparison between groups was performed by the χ^2^ test. The sample with missing data were deleted. The confidence level was α = 0.05. All statistical analyses were performed using SAS v. 9.4 (SAS Institute, Cary, North Carolina) and R v. 4.0.3 (R Foundation for Statistical Computing, Vienna, Austria).

## Results

### Characteristics of the study population

Of 571 patients treated for ACS and hospitalized in our hospital, 498 patients were included in the analysis finally. 9 patients with repeated data collection and 2 patients died were excluded. We also excluded patients with variables missing, including weight (n = 3), Gensini score (n = 6), congestive heart failure (n = 30), Mb (n = 8), LVEF (n = 3), CK-MB (n = 7), BNP (n = 1), and TC (n = 4). The flowchart of the selection is described in Fig. 1. Lost to follow-up rate was 3%.

The final data set for analyses consisted of 370 men and 128 women, the mean age for all ACS patients was 64.37 (± 10.02 SD) in Table 1. Of these 76 (15.26%) had a NSTEMI, 122 (24.50%) had STEMI and 300 (60.24%) had UA. Of the whole group 176 (35.34%) patients had diabetes, 289 (58.03%) patients had hypertension and 346 (69.48%) patients had other complications. Average length of hospital stay was 7 days. 83 ACS patients were readmitted within six months, with a readmission rate of 16.67%. No significant difference was found in the studied variables between training cohort and validation cohort (Supplement Table 1).

**Table 1:** Characteristics of ACS patients readmitted and not readmitted at 6 months

Variables	Total (n = 498)	Non-readmission group (n = 415)	Readmission group (n = 83)	Statistics	***P***
Age, years, Mean ± SD	64.37 ± 10.02	63.97 ± 9.74	66.33 ± 11.17	t=-1.96	0.051
Gender, n (%)				χ^2^ = 2.431	0.119
Male	370 (74.30)	314 (75.66)	56 (67.47)		
Female	128 (25.70)	101 (24.34)	27 (32.53)		
Height, m, Mean ± SD	1.68 ± 0.08	1.68 ± 0.08	1.66 ± 0.09	t = 1.91	0.057
Weight, kg, Mean ± SD	69.90 ± 11.92	70.24 ± 11.57	68.21 ± 13.46	t = 1.42	0.157
BMI, kg/m^2^, Mean ± SD	24.72 ± 3.27	24.77 ± 3.25	24.51 ± 3.37	t = 0.66	0.511
Smoking history, n (%)				χ^2^ = 1.262	0.261
No	260 (52.21)	212 (51.08)	48 (57.83)		
Yes	238 (47.79)	203 (48.92)	35 (42.17)		
Alcohol drinking history, n (%)				χ^2^ = 0.556	0.456
No	312 (62.65)	257 (61.93)	55 (66.27)		
Yes	186 (37.35)	158 (38.07)	28 (33.73)		
Gensini score, M (Q_1_, Q_3_)	40.00 (24.00, 60.00)	40.00 (24.00, 60.00)	39.00 (23.00, 61.00)	Z=-0.299	0.765
ACS type, n (%)				χ^2^ = 13.912	< 0.001
NSTEMI	76 (15.26)	61 (14.70)	15 (18.07)		
STEMI	122 (24.50)	115 (27.71)	7 (8.43)		
UA	300 (60.24)	239 (57.59)	61 (73.49)		
Number of diseased vessels, n (%)				χ^2^ = 1.508	0.470
One	181 (36.35)	153 (36.87)	28 (33.73)		
Two	142 (28.51)	121 (29.16)	21 (25.30)		
Three	175 (35.14)	141 (33.98)	34 (40.96)		
Treatment, n (%)				χ^2^ = 48.454	< 0.001
PCI	444 (89.16)	388 (93.49)	56 (67.47)		
Others ^a^	54 (10.84)	27 (6.51)	27 (32.53)		
Diabetes mellitus, n (%)				χ^2^ = 3.719	0.054
No	322 (64.66)	276 (66.51)	46 (55.42)		
Yes	176 (35.34)	139 (33.49)	37 (44.58)		
Hypertension, n (%)				χ^2^ = 5.740	0.017
No	209 (41.97)	184 (44.34)	25 (30.12)		
Yes	289 (58.03)	231 (55.66)	58 (69.88)		
Comorbidities ^b^, n (%)				χ^2^ = 4.735	0.030
No	152 (30.52)	135 (32.53)	17 (20.48)		
Yes	346 (69.48)	280 (67.47)	66 (79.52)		
HGB, g/L, Mean ± SD	136.85 ± 17.35	137.36 ± 16.76	134.30 ± 19.98	t = 1.31	0.194
WBC, 10^9^/L, M (Q_1_, Q_3_)	7.27 (6.02, 9.16)	7.28 (5.98, 9.34)	7.15 (6.28, 8.70)	Z=-0.590	0.555
PLT, 10^9^/L, Mean ± SD	215.33 ± 57.40	217.07 ± 58.17	206.61 ± 52.84	t = 1.52	0.130
NEUT, 10^9^/L, M (Q_1_, Q_3_)	4.42 (3.37, 6.33)	4.41 (3.36, 6.47)	4.62 (3.47, 5.69)	Z=-0.134	0.893
Cr, µmol/L, M (Q_1_, Q_3_)	78.00 (68.00, 92.00)	78.00 (68.00, 91.00)	79.00 (67.00, 96.00)	Z = 0.931	0.352
SUA, µmol/L, Mean ± SD	351.96 ± 97.42	346.17 ± 91.58	380.89 ± 118.99	t=-2.51	0.014
TC, mmol/L, M (Q_1_, Q_3_)	3.78 (3.13, 4.48)	3.78 (3.18, 4.44)	3.78 (3.04, 4.74)	Z = 0.417	0.677
TG, mmol/L, M (Q_1_, Q_3_)	1.41 (0.99, 2.05)	1.41 (0.99, 2.02)	1.51 (1.00, 2.34)	Z = 1.026	0.305
HDL, mmol/L, Mean ± SD	1.01 (0.84, 1.17)	1.01 (0.85, 1.17)	0.97 (0.80, 1.17)	Z=-0.791	0.429
LDL, mmol/L, M (Q_1_, Q_3_)	2.28 (1.79, 3.06)	2.29 (1.79, 3.06)	2.23 (1.63, 3.11)	Z=-0.310	0.757
CK-MB, µg/L, M (Q_1_, Q_3_)	14.45 (11.20, 30.60)	14.30 (11.00, 34.50)	14.80 (11.50,21.70)	Z=-0.719	0.472
BNP, pg/mL, M (Q_1_, Q_3_)	147.00 (58.00, 810.00)	147.00 (59.00, 776.00)	151.00 (57.00,1243.00)	Z = 1.083	0.279
cTnI, ng/ml, M (Q_1_, Q_3_)	0.02 (0.01, 0.47)	0.02 (0.01, 0.57)	0.02 (0.01, 0.22)	Z=-0.532	0.595
Mb, ng/ml, M (Q_1_, Q_3_)	31.65 (21.00, 64.40)	31.70 (21.00, 66.30)	31.50 (21.00, 59.90)	Z=-0.716	0.474
LVEF, %, n (%)				χ^2^ = 1.376	0.241
<60	153 (30.72)	123 (29.64)	30 (36.14)		
≥60	345 (69.28)	292 (70.36)	53 (63.86)		
eGFR, ml/min, M (Q_1_, Q_3_)	92.42 (77.15, 106.62)	92.98 (78.76, 106.62)	83.42 (72.65, 110.87)	Z=-1.622	0.105
Length of stay, days, M (Q_1_, Q_3_)	7.00 (5.00, 10.00)	7.00 (5.00, 10.00)	8.00 (5.00, 14.00)	Z = 1.271	0.204
Adverse events occurred during hospitalization ^c^, n (%)				χ^2^ = 20.413	< 0.001
No	515 (93.47)	437 (95.62)	78 (82.98)		
Yes	36 (6.53)	20 (4.38)	16 (17.02)		
Clopidogrel, n (%)				χ^2^ = 0.796	0.372
No	457 (82.94)	382 (83.59)	75 (79.79)		
Yes	94 (17.06)	75 (16.41)	19 (20.21)		
Calcium channel blockers, n (%)				χ^2^ = 0.021	0.885
No	431 (78.22)	358 (78.34)	73 (77.66)		
Yes	120 (21.78)	99 (21.66)	21 (22.34)		
ACEI/ARB, n (%)				χ^2^ = 0.113	0.737
No	435 (78.95)	362 (79.21)	73 (77.66)		
Yes	116 (21.05)	95 (20.79)	21 (22.34)		
Statins, n (%)				χ^2^ = 4.951	0.026
No	63 (11.43)	46 (10.07)	17 (18.09)		
Yes	488 (88.57)	411 (89.93)	77 (81.91)		
Diuretics, n (%)				χ^2^ = 2.960	0.085
No	471 (85.48)	396 (86.65)	75 (79.79)		
Yes	80 (14.52)	61 (13.35)	19 (20.21)		

ACS: acute coronary syndrome; SD: standard deviation; BMI: body mass index; NSTEMI: non-ST-segment elevation myocardial infarction; STEMI: ST-segment elevation myocardial infarction; UA: unstable angina; PCI: percutaneous coronary intervention; HGB: hemoglobin; WBC: white blood cells; PLT: blood platelet; NEUT: neutrophils; Cr: creatinine; SUA: serum uric acid; TC: total cholesterol; TG: triglyceride; HDL: high-density lipoprotein cholesterol; LDL: low-density lipoprotein cholesterol; CK-MB: creatine kinase-MB; BNP: brain natriuretic peptide; cTnI: cardiac troponin I; Mb: myoglobin; LVEF: left ventricular ejection fraction; eGFR: glomerular filtration rate.

^a^ Other treatments include cardiovascular primary prevention, cardiovascular secondary prevention, thrombolysis, and drugs.

^b^ Comorbidities include gout, congestive heart failure, end-stage renal disease, chronic obstructive pulmonary disease, peripheral vascular disease, hypothyroidism, depression, etc.

^c^ Adverse events that occurred during hospitalization include cardiac arrest, coronary dissection, coronary perforation, acute kidney injury, major bleeding, cardiogenic shock, acute respiratory failure, etc.

### Predictors of readmission within six months in ACS patients

The results of univariate analysis in Table 1 showed that there were significant differences between the non-readmission group and the readmission group in ACS type (*P* < 0.001), treatment (*P* < 0.001), hypertension (*P* = 0.017), comorbidities (*P* = 0.030), SUA (*P* = 0.014), incidence of adverse events during hospitalization (*P* < 0.001) and statins (*P* = 0.026).

The results of the multivariate logistic analysis are presented in Table 2. Patients with STEMI and UA had a 4.00-fold [OR = 4.00, 95%CI: (1.53–10.44)] and 4.78-fold [OR = 4.78, 95%CI: (2.05–11.14)] higher risk of readmission within six months than those with NSTEMI in Model 2 (multivariate logistic regression adjusted for age, BMI, gender, smoking history, alcohol drinking history, and Gensini score), respectively. Use of other treatment modalities was associated with increased risk of readmission within six months compared with ACS patients treated with PCI [OR = 7.37, 95%CI: (3.95–13.75)]. Patients with hypertension had a 1.81 times higher risk of readmission within six months than those without hypertension [OR = 1.81, 95%CI: (1.08–3.05)]. The level of SUA [OR = 1.01, 95%CI: (1.01–1.01)] and length of stay [OR = 1.05, 95%CI: (1.01–1.08)] were associated to the risk of readmission within six months. Patients taking statins were associated with a reduced risk of readmission within six months compared with patients not taking statins [OR = 0.48, 95%CI: (0.25–0.91)]. Patients with adverse events during hospitalization had a 3.12 times higher risk of readmission within six months than patients without adverse events during hospitalization [OR = 4.12, 95%CI: (1.83–9.28)].

**Table 2:** logistic analysis to explore predictors associated with readmission within six months of ACS patients

	Model 1		Model 2	
Variables	Odds Ratio (95% CI)	*** P***	Odds Ratio (95% CI)	*** P***
ACS type				
NSTEMI	Ref		Ref	
STEMI	4.04 (1.56–10.44)	0.004	4.00 (1.53–10.44)	0.005
UA	4.19 (1.86–9.46)	< 0.001	4.78 (2.05–11.14)	< 0.001
Treatment				
PCI	Ref		Ref	
Others	6.93 (3.80-12.66)	< 0.001	7.37 (3.95–13.75)	< 0.001
Hypertension				
No	Ref		Ref	
Yes	1.85 (1.11–3.07)	0.018	1.81 (1.08–3.05)	0.025
Complications				
No	Ref		Ref	
Yes	1.87 (1.06–3.31)	0.031	1.76 (0.98–3.15)	0.057
SUA	1.003 (1.001–1.006)	0.004	1.01 (1.01–1.01)	< 0.001
Length of stay	1.04 (1.003–1.073)	0.034	1.05 (1.01–1.08)	0.010
Statins				
No	Ref		Ref	
Yes	0.48 (0.26–0.91)	0.028	0.48 (0.25–0.91)	0.024
Adverse events occurred during hospitalization				
No	Ref		Ref	
Yes	4.21 (1.91–9.29)	< 0.001	4.12 (1.83–9.28)	< 0.001

Ref: reference; CI: confidence interval; NSTEMI: non-ST-segment elevation myocardial infarction; STEMI: ST-segment elevation myocardial infarction; UA: unstable angina; PCI: percutaneous coronary intervention; SUA: serum uric acid; Model 1: univariate logistic regression; Model 2: multivariate logistic regression adjusted for age, BMI, gender, smoking history, alcohol drinking history, and Gensini score

### Development of a prediction model for readmission within 6 months after treatment in ACS patients

Predictors included ACS type, treatment, hypertension, SUA, length of stay, statins, and adverse events occurred during hospitalization were used to form a six-month readmission prediction model for ACS patients. The formula is as follows.

$$\begin{gathered} logit(p)= - 5.20+0.49 * hypertension \\ +ACS+0.003 * SUA+0.8 * length\;of\;stay\; \\ +2.05 * PCI - 0.46 * statins \\ +1.31 * adverse{\text{ }}events{\text{ }}occurred{\text{ }}during{\text{ }}hospitalization \\ \end{gathered}$$

Hypertension (No = 0, Yes = 1); ACS (STEMI = 0, NSTEMI = 1.64, UA = 1.85); statins (No = 0, Yes = 1); adverse events occurred during hospitalization (No = 0, Yes = 1).

**Fig. 2:**
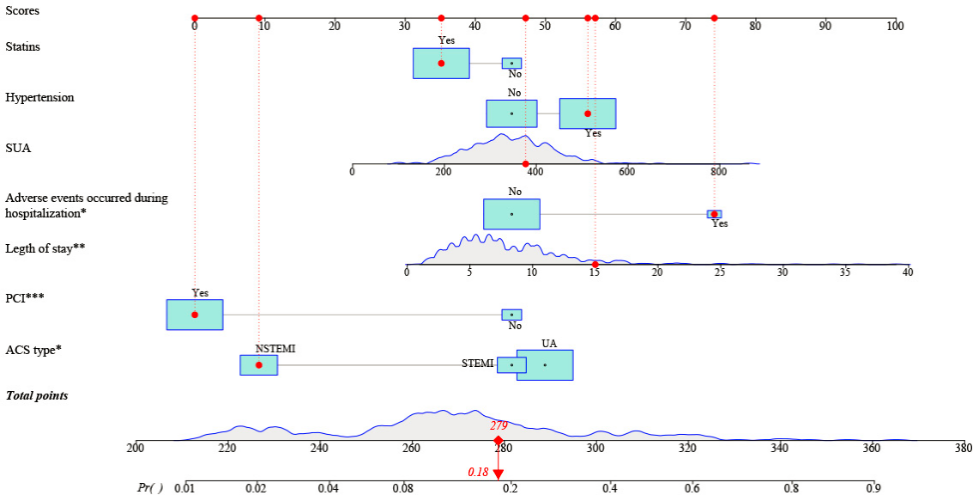
A six-month readmission estimation nomogram. *: *P* < 0.05; **: *P* < 0.01; ***: *P* < 0.001; SUA: serum uric acid; PCI: percutaneous coronary intervention; ACS: acute coronary syndrome

And a nomogram for readmission within 6 months was also developed (Fig. 2).

### Performance of the prediction model

**Fig. 3:**
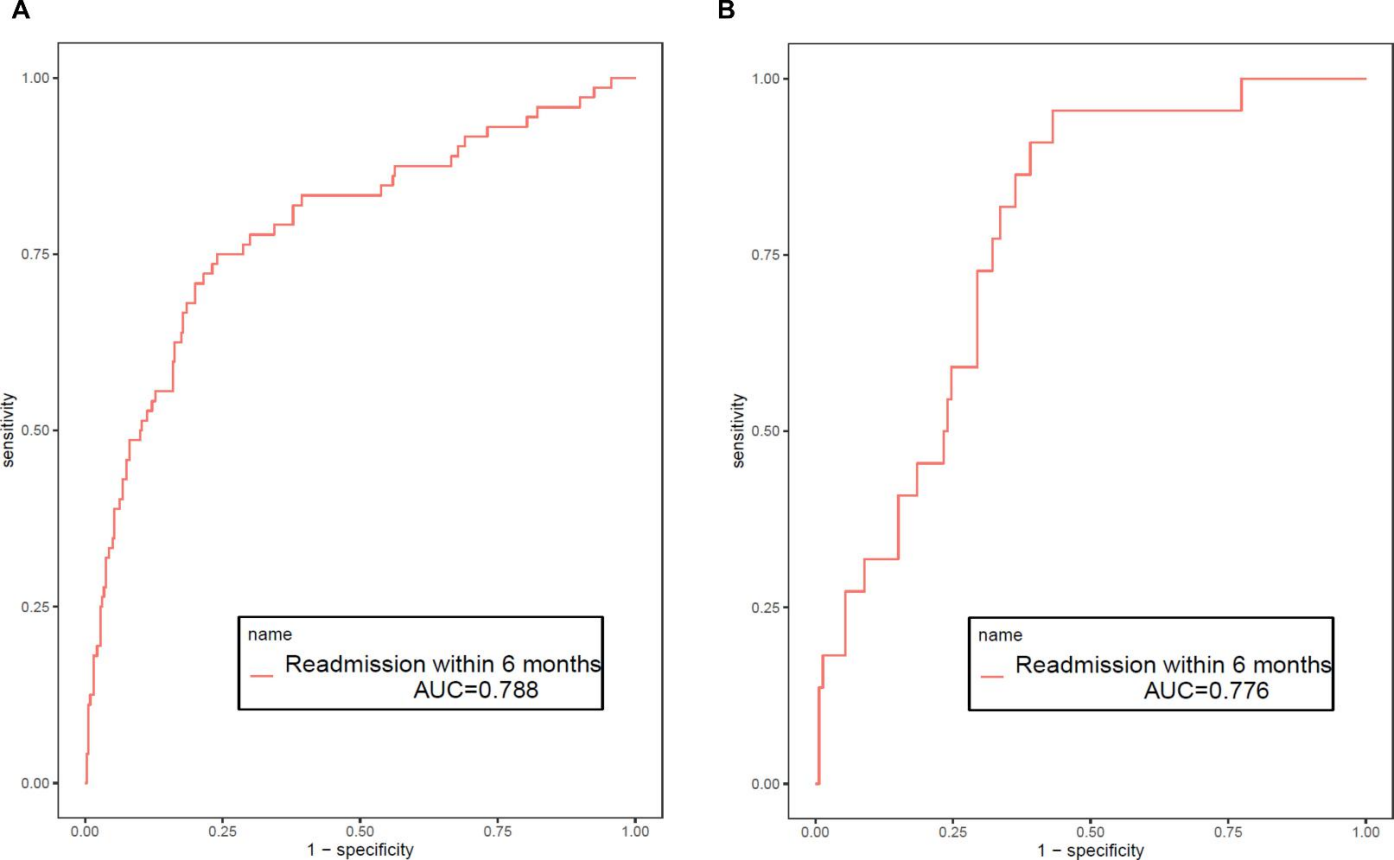
ROC curves of training cohort (**A**) and validation cohort (**B**)

**Fig. 4:**
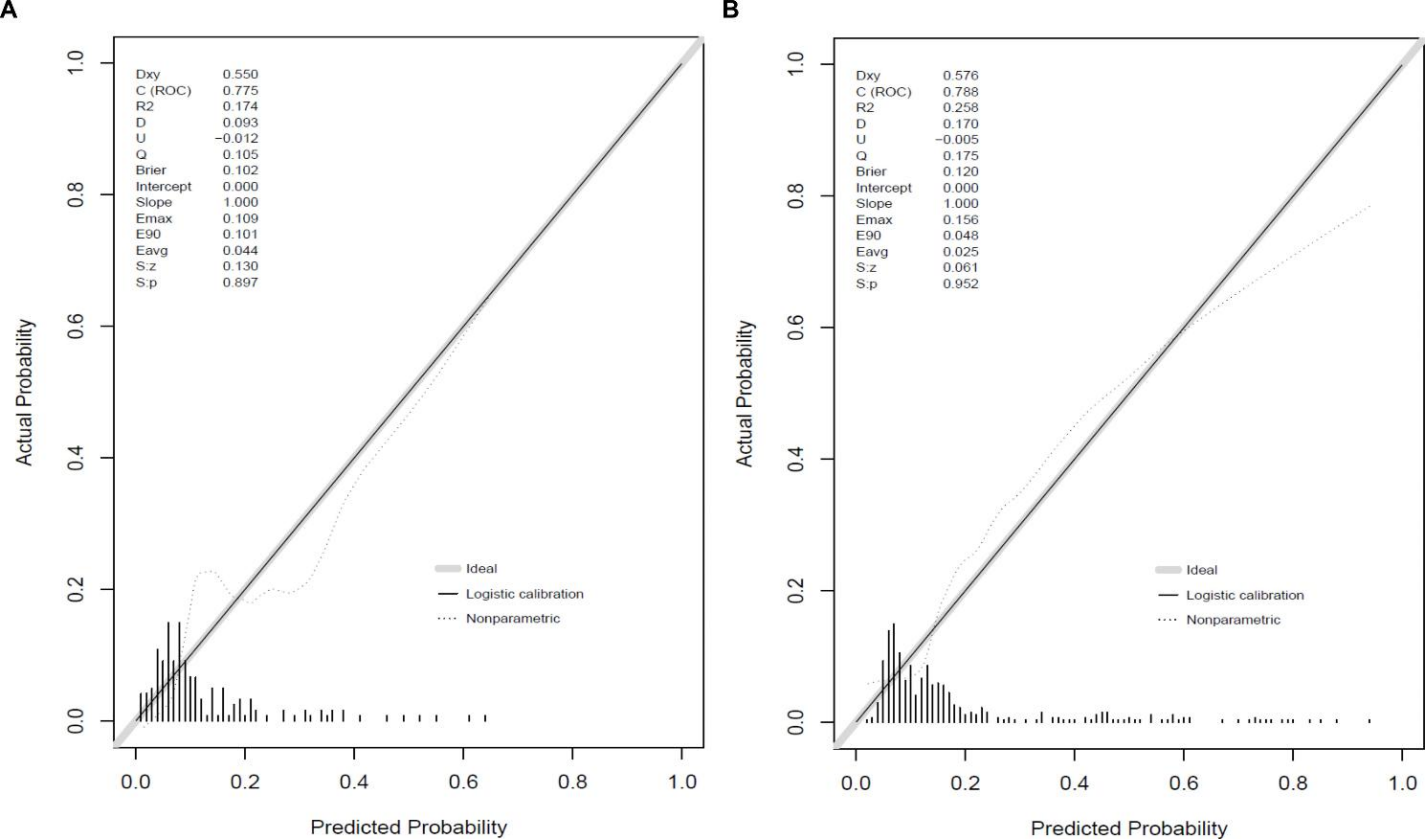
Calibration curves of training cohort (**A**) and validation cohort (**B**)

**Fig. 5:**
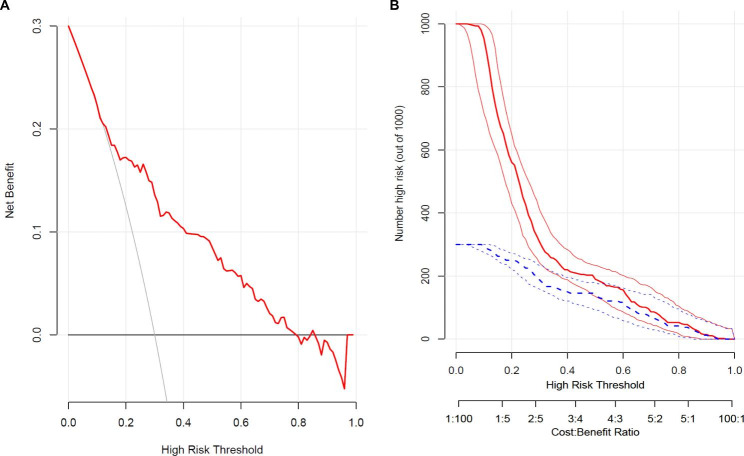
(**A**) Decision curve analysis for the nomogram; (**B**) clinical impact curve for the nomogram

In the training cohort, the AUC was 0.788 (95%CI: 0.735–0.878), accuracy was 0.805 (95%CI: 0.760–0.845), specificity was 0.840 (95%CI: 0.798–0.883) and sensitivity was 0.639 (95%CI: 0.519–0.760) in Table 3. And the AUC, accuracy, specificity and sensitivity were 0.775 (95%CI: 0.686–0.865), 0.685 (95%CI: 0.603–0.758), 0.701 (95%CI: 0.621–0.780), and 0.591 (95%CI: 0.385–0.796) in the validation cohort, respectively. The value of cut off was 0.195. Figure 3 A and 3B show the ROC of training cohort and validation cohort. Calibration curves in Fig. 4 A and 4B showed the good calibration of the prediction model. Besides, the clinical impact curve for the model was also visually indicated that nomogram conferred high clinical net benefit and confirmed the clinical value of this model (Fig. 5 A and 5B). Finally, this model was internally validated using bootstrapping resampling of the construction data set (with 1000 bootstrap samples per model). Moreover, the C-index for this nomogram was 0.758, which suggested high accuracy.

**Table 3:** Predictive performance of training cohort and validation cohort

	AUC (95%CI)	Accuracy (95%CI)	Specificity (95%CI)	Sensitivity (95%CI)	Cut off
Training cohort	0.788 (0.735–0.878)	0.805 (0.760–0.845)	0.840 (0.798–0.883)	0.639 (0.519–0.760)	0.195
Validation cohort	0.775 (0.686–0.865)	0.685 (0.603–0.758)	0.701 (0.621–0.780)	0.591 (0.385–0.796)	0.195
C-index					
Internal validation (with 1000 bootstrap samples per model)				0.758	

AUC: area under the curve; CI: confidence interval

We took a patient in the cohort as an example, took statins, had hypertension, level of SUA was 380 µmol/L, hospitalization length of 15 days, had adverse events during hospitalization, received PCI, ACS type was NSTEMI, the overall score calculated by nomogram was 279 and the probability of six-month readmission was 0.18 (Fig. 2).

## Discussion

ACS is an important cause of death from cardiovascular disease. Readmission is a common, costly, and preventable adverse event in ACS patients. Although the survival rate of ACS patients after hospitalization has improved, early readmission is still a common phenomenon, which significantly increases the economic burden of patients and seriously affects the quality of patients’ life. It is important to explore and identify these patients at high risk of readmission and the predictors associated with readmission, so that proactive secondary prevention measures could be taken to reduce ACS readmissions, improve short- and long-term outcomes, and reduce economic burden. In this study, we developed a prediction model and generated nomogram for six-month readmissions in patients with ACS using ACS type, treatment, hypertension, SUA, length of stay, statins, and adverse events occurred during hospitalization. And the prediction model has been internally verified to have good predictive performance.

To date, there is few prediction model to identify the risk of ACS readmission. Albuquerque RN et al. proposed a prediction model for ACS hospital readmission using drug therapy adherence, stress, number of years smoked and the use of services in primary healthcare units [9]. But there was no visualization of the prediction model. In our study, using regression equations for prediction, especially the nomogram has the advantages of being more intuitive, vivid and simpler than traditional prediction methods. To the best of our knowledge, we developed the first prediction model and nomogram for readmission within six months of patients diagnosed with ACS who were discharged after treatment. Our nomogram used seven predictors that were easily available during patient admission. The nomogram has non-invasive clinical features [10] and can immediately and reliably estimate the risk of readmission within six months of ACS patients. The estimate can guide clinicians in counseling patients and/or families, early identification of patients at high risk of readmission, and other treatments.

The predictors for 6-month readmission of ACS patients that we explored in this study were ACS type, treatment, hypertension, SUA, length of stay, statins, and adverse events occurred during hospitalization. Several studies were also consistent with our findings. The development and popularization of PCI has improved the prognosis of ACS patients and has been widely used in clinical practice [15]. Study showed PCI associated with lower 30-day readmission rates and costs in ACS patients [16]. And a retrospective analysis of patients who had undergone coronary angiogram in the U.S. found that the 30-day readmission rate was lower in the PCI group than in the non-PCI group [17]. However, PCI could lead to complications, such as coronary dissection [18], coronary perforation [19], massive bleeding [20], stent thrombosis [21], etc. The occurrence of these adverse events may increase the risk of readmission. A cohort study from the US Readmission Database 2014 showed that length of stay ≥ 5 days and adverse events during admission, such as acute kidney injury, major bleeding were predictors of increased readmission [22]. Secondary prevention drugs such as statins could reduce chest pain and reinfarction, thereby reducing the possibility of rehospitalization [23]. Sreenivasan et al. [24] studied ACS patients of the National Readmission Database found that medical comorbidities (hypertension) were the independent predictors of increased readmission risk. The study by Stamp et al. [25] showed that as uric acid levels increased, adverse outcomes including the risk of readmission also increased. Some of these factors are modifiable, depending on lifestyle changes, investment in nursing care, and health services that provide care after discharge.

The strength of this study was that there was still a lack of clinically convenient and practical prediction models for identifying high-risk patients with ACS readmission in China. This study used common indicators in clinical diagnosis and treatment to establish a prediction model for predicting readmission of ACS patients within six months. Our nomogram showed good predictive performance, which could help clinicals identify patients who need active attention and frequent follow-up early, and guide the management of patients. There were a few limitations in our study. First, the study was a retrospective cohort study, and physical activity, psychological factors, and socioeconomic factors on admission and after discharge were not collected, which may also be influencing factors for readmission in ACS patients. Second, although this study was internally validated, there was a lack of independent external data to validate the model. And all patients in this study were selected from China, which may limit the global generalizability of our findings.

## Conclusion

This study used seven easily acquired clinical variables (ACS type, treatment, hypertension, SUA, length of stay, statins, and adverse events occurred during hospitalization) to develop a prediction model for readmission within six months of ACS patients, and developed a nomogram, which was internally validated to predict well. The results of this study could be used to inform the prognosis and intervention design of patients with high-risk ACS for 6-month readmission.

## Electronic supplementary material

Below is the link to the electronic supplementary material.

Supplementary Material 1: Comparison of characteristics in the training cohort and validation cohort of ACS patients

## Data Availability

The datasets during and/or analysed during the current study are available from the corresponding author on reasonable request.
