# Supplementary material for: A nomogram for predicting the readmission within 6 months after treatment in patients with acute coronary syndrome
Source: BMC Cardiovasc Disord. 2022 Oct 26;22:448. doi: 10.1186/s12872-022-02873-6 (PMC9608930; doi:10.1186/s12872-022-02873-6)
Supplement: Supplementary file 1 — Supplementary Material 1: Comparison of characteristics in the training cohort and validation cohort of ACS patients [file 12872_2022_2873_MOESM1_ESM.docx]

Supplement Table 1 Comparison of characteristics in the training cohort and validation cohort of ACS patients

| Variables | Total (n=498) | Validation cohort (n=149) | Training cohort (n=349) | Statistics | *P* |
| --- | --- | --- | --- | --- | --- |
| Age, years, Mean ± SD | 64.37 ± 10.02 | 64.83 ± 9.18 | 64.17 ± 10.37 | t=0.67 | 0.504 |
| Gender, n (%) |  |  |  | χ^2^=0.025 | 0.875 |
| Male | 370 (74.30) | 110 (73.83) | 260 (74.50) |  |  |
| Female | 128 (25.70) | 39 (26.17) | 89 (25.50) |  |  |
| Height, m, Mean ± SD | 1.68 ± 0.08 | 1.68 ± 0.08 | 1.68 ± 0.08 | t=0.05 | 0.963 |
| Weight, kg, Mean ± SD | 69.90 ± 11.92 | 69.87 ± 12.09 | 69.92 ± 11.86 | t=-0.05 | 0.962 |
| BMI, kg/m^2^, Mean ± SD | 24.72 ± 3.27 | 24.71 ± 3.37 | 24.73 ± 3.23 | t=-0.05 | 0.957 |
| Smoking history, n (%) |  |  |  | χ^2^=0.024 | 0.877 |
| No | 260 (52.21) | 77 (51.68) | 183 (52.44) |  |  |
| Yes | 238 (47.79) | 72 (48.32) | 166 (47.56) |  |  |
| Alcohol drinking history, n (%) |  |  |  | χ^2^=0.774 | 0.379 |
| No | 312 (62.65) | 89 (59.73) | 223 (63.90) |  |  |
| Yes | 186 (37.35) | 60 (40.27) | 126 (36.10) |  |  |
| Gensini score, M (Q_1_, Q_3_) | 40.00 (24.00,60.00) | 38.00 (24.00,58.00) | 42.00 (24.00,62.00) | Z=-0.994 | 0.320 |
| ACS type, n (%) |  |  |  | χ^2^=1.263 | 0.532 |
| NSTEMI | 76 (15.26) | 22 (14.77) | 54 (15.47) |  |  |
| STEMI | 122 (24.50) | 32 (21.48) | 90 (25.79) |  |  |
| UA | 300 (60.24) | 95 (63.76) | 205 (58.74) |  |  |
| Number of diseased vessels, n (%) |  |  |  | χ^2^=3.474 | 0.176 |
| One | 181 (36.35) | 54 (36.24) | 127 (36.39) |  |  |
| Two | 142 (28.51) | 35 (23.49) | 107 (30.66) |  |  |
| Three | 175 (35.14) | 60 (40.27) | 115 (32.95) |  |  |
| Treatment, n (%) |  |  |  | χ^2^=0.002 | 0.961 |
| PCI | 444 (89.16) | 133 (89.26) | 311 (89.11) |  |  |
| Others ^a^ | 54 (10.84) | 16 (10.74) | 38 (10.89) |  |  |
| Diabetes mellitus, n (%) |  |  |  | χ^2^=0.296 | 0.586 |
| No | 322 (64.66) | 99 (66.44) | 223 (63.90) |  |  |
| Yes | 176 (35.34) | 50 (33.56) | 126 (36.10) |  |  |
| Hypertension, n (%) |  |  |  | χ^2^=3.573 | 0.059 |
| No | 209 (41.97) | 53 (35.57) | 156 (44.70) |  |  |
| Yes | 289 (58.03) | 96 (64.43) | 193 (55.30) |  |  |
| Comorbidities ^b^, n (%) |  |  |  | χ^2^=0.560 | 0.454 |
| No | 152 (30.52) | 49 (32.89) | 103 (29.51) |  |  |
| Yes | 346 (69.48) | 100 (67.11) | 246 (70.49) |  |  |
| HGB, g/L, Mean ± SD | 136.85 ± 17.35 | 137.38 ± 17.84 | 136.62 ± 17.16 | t=0.45 | 0.656 |
| WBC, 10^9^/L, M (Q_1_, Q_3_) | 7.27 (6.02,9.16) | 7.25 (5.90,8.90) | 7.31 (6.05,9.24) | Z=-0.547 | 0.584 |
| PLT, 10^9^/L, Mean ± SD | 215.33 ± 57.40 | 217.64 ± 54.61 | 214.34 ± 58.60 | t=0.59 | 0.557 |
| NEUT, 10^9^/L, M (Q_1_, Q_3_) | 4.42 (3.37,6.33) | 4.29 (3.28,5.90) | 4.47 (3.40,6.41) | Z=-0.940 | 0.347 |
| Cr, μmol/L, M (Q_1_, Q_3_) | 78.00 (68.00,92.00) | 80.00 (68.00,96.00) | 78.00 (68.00,90.00) | Z=1.064 | 0.287 |
| SUA, μmol/L, Mean ± SD | 351.96 ± 97.42 | 364.28 ± 109.13 | 346.70 ± 91.63 | t=1.72 | 0.086 |
| TC, mmol/L, M (Q_1_, Q_3_) | 3.91 ± 1.10 | 3.95 ± 1.13 | 3.90 ± 1.09 | t=0.55 | 0.582 |
| TG, mmol/L, M (Q_1_, Q_3_) | 1.41 (0.99,2.05) | 1.51 (1.05,2.10) | 1.38 (0.99,2.04) | Z=0.904 | 0.366 |
| HDL, mmol/L, Mean ± SD | 1.04 ± 0.28 | 1.02 ± 0.30 | 1.05 ± 0.28 | t=-0.91 | 0.363 |
| LDL, mmol/L, M (Q_1_, Q_3_) | 2.28 (1.79,3.06) | 2.37 (1.81,3.13) | 2.26 (1.76,3.03) | Z=0.518 | 0.605 |
| CK-MB, μg/L, M (Q_1_, Q_3_) | 14.45 (11.20,30.60) | 14.90 (11.60,33.20) | 14.20 (11.00,29.60) | Z=0.889 | 0.374 |
| BNP, pg/mL, M (Q_1_, Q_3_) | 147.00 (58.00,810.00) | 140.00 (56.00,810.00) | 151.00 (62.00,782.00) | Z=-0.089 | 0.929 |
| cTnI, ng/ml, M (Q_1_, Q_3_) | 0.02 (0.01,0.47) | 0.02 (0.01,0.43) | 0.02 (0.01,0.49) | Z=-0.207 | 0.836 |
| Mb, ng/ml, M (Q_1_, Q_3_) | 31.65 (21.00,64.40) | 30.60 (21.00,61.50) | 31.80 (21.00,64.40) | Z=-0.218 | 0.827 |
| LVEF, %, n (%) |  |  |  | χ^2^=0.067 | 0.795 |
| <60 | 153 (30.72) | 47 (31.54) | 106 (30.37) |  |  |
| ≥60 | 345 (69.28) | 102 (68.46) | 243 (69.63) |  |  |
| eGFR, ml/min, M (Q_1_, Q_3_) | 92.14 ± 26.36 | 89.54 ± 27.50 | 93.25 ± 25.82 | t=-1.44 | 0.150 |
| Length of stay, days, M (Q_1_, Q_3_) | 7.00 (5.00,10.00) | 7.00 (5.00,11.00) | 7.00 (5.00,10.00) | Z=0.430 | 0.667 |
| Adverse events occurred during hospitalization ^c^, n (%) |  |  |  | χ^2^=0.026 | 0.873 |
| No | 470 (94.38) | 141 (94.63) | 329 (94.27) |  |  |
| Yes | 28 (5.62) | 8 (5.37) | 20 (5.73) |  |  |
| Clopidogrel, n (%) |  |  |  | χ^2^=1.303 | 0.254 |
| No | 406 (81.53) | 126 (84.56) | 280 (80.23) |  |  |
| Yes | 92 (18.47) | 23 (15.44) | 69 (19.77) |  |  |
| Calcium channel blockers, n (%) |  |  |  | χ^2^=1.656 | 0.198 |
| No | 386 (77.51) | 110 (73.83) | 276 (79.08) |  |  |
| Yes | 112 (22.49) | 39 (26.17) | 73 (20.92) |  |  |
| ACEI/ARB, n (%) |  |  |  | χ^2^=1.767 | 0.184 |
| No | 396 (79.52) | 113 (75.84) | 283 (81.09) |  |  |
| Yes | 102 (20.48) | 36 (24.16) | 66 (18.91) |  |  |
| Statins, n (%) |  |  |  | χ^2^=1.733 | 0.188 |
| No | 59 (11.85) | 22 (14.77) | 37 (10.60) |  |  |
| Yes | 439 (88.15) | 127 (85.23) | 312 (89.40) |  |  |
| Diuretics, n (%) |  |  |  | χ^2^=1.268 | 0.260 |
| No | 434 (87.15) | 126 (84.56) | 308 (88.25) |  |  |
| Yes | 64 (12.85) | 23 (15.44) | 41 (11.75) |  |  |

ACS: acute coronary syndrome; SD: standard deviation; BMI: body mass index; NSTEMI: non-ST-segment elevation myocardial infarction; STEMI: ST-segment elevation myocardial infarction; UA: unstable angina; PCI: percutaneous coronary intervention; HGB: hemoglobin; WBC: white blood cells; PLT: blood platelet; NEUT: neutrophils; Cr: creatinine; SUA: serum uric acid; TC: total cholesterol; TG: triglyceride; HDL: high-density lipoprotein cholesterol; LDL: low-density lipoprotein cholesterol; CK-MB: creatine kinase-MB; BNP: brain natriuretic peptide; cTnI: cardiac troponin I; Mb: myoglobin; LVEF: left ventricular ejection fraction; eGFR: glomerular filtration rate.

^a.^ Other treatments include cardiovascular primary prevention, cardiovascular secondary prevention, thrombolysis, and drugs.

^b.^ Comorbidities include gout, congestive heart failure, end-stage renal disease, chronic obstructive pulmonary disease, peripheral vascular disease, hypothyroidism, depression, etc.

^c.^ Adverse events that occurred during hospitalization include cardiac arrest, coronary dissection, coronary perforation, acute kidney injury, major bleeding, cardiogenic shock, acute respiratory failure, etc.
